# Editorial: Role of Protein Palmitoylation in Synaptic Plasticity and Neuronal Differentiation

**DOI:** 10.3389/fnsyn.2020.00027

**Published:** 2020-07-09

**Authors:** Akira Yoshii, William N. Green

**Affiliations:** ^1^Department of Anatomy and Cell Biology, Pediatrics, Neurology, University of Illinois at Chicago, Chicago, IL, United States; ^2^Department of Neurobiology, University of Chicago, Chicago, IL, United States; ^3^The Marine Biological Laboratory, Woods Hole, MA, United States

**Keywords:** palmitoylation and depalmitoylation, synaptic plasticity, axonal growth, lysosome, neurodegenerative disease, neuronal ceroid lipofuscinoses (NCL), Huntington disease

Protein palmitoylation, the reversible addition of palmitate to proteins, is a dynamic post-translational modification. Both membrane (e.g., channels, transporters, and receptors) and cytoplasmic proteins (e.g., cell adhesion, scaffolding, cytoskeletal, and signaling molecules) are substrates. In mammals, palmitoylation is mediated by 23-24 palmitoyl acyltransferases (PATs), also called ZDHHCs for their catalytic aspartate-histidine-histidine-cysteine (DHCC) domain. PATs are integral membrane proteins found in cellular membranes. In the palmitoylation cycle, palmitate is removed by the depalmitoylation enzymes, acyl palmitoyl transferases (APT1 and 2), and α/β Hydrolase domain-containing protein 17 (ABHD17A-C). These are cytoplasmic proteins that are targeted to membranes where they are substrates for PATs. The second class of depalmitoylating enzymes are palmitoyl thioesterases, PPT1 and 2, discovered through their association with infantile neuronal ceroid lipofuscinosis. These are secreted proteins found in the lumen of intracellular organelles, primarily lysosomes, where their function as depalmitoylating enzymes is unclear.

Our understanding of protein palmitoylation is still in its infancy, with the most basic questions still to be addressed. Have all of the proteins involved in the process of palmitoylation been identified? What are the molecular details for how palmitoylation and depalmitoylation occurs and how substrates are targeted to and bind to PATs and deplamitoylating enzymes? What roles do the different PATs and deplamitoylating enzymes play? How is palmitoylation tied to and regulated by lipid metabolism and other metabolic pathways? How do other post-translational modifications at overlapping sites on proteins, such as phosphorylation and nitrosylation, regulate palmitoylation and vice versa?

One area where progress is being made is characterizing the different functions of palmitoylation in neurons. Here several groups have investigated the role of palmitoylation in different cell biological processes involved in axon growth, synapse formation, synaptic plasticity, and lysosomal function at synapses. Of special interest are the links these processes have to neurodegenerative diseases, in particular infantile neuronal ceroid lipofuscinosis and Huntington's Disease.

Matt et al. review the dynamic cycling of protein palmitoylation and its role in activity-dependent synapse formation. Palmitoylation of postsynaptic proteins ranges from neurotransmitter receptors such as the N-methyl-D-aspartate receptor (NMDAR) and α-amino-3-hydroxy-5-methyl-4-isoxazole propionic acid receptor (AMPAR) to scaffolding proteins such as postsynaptic density protein 95 (PSD-95). Especially, PSD-95 palmitoylation is a critical regulator of many aspects of synaptic plasticity. For example, another study from the same group demonstrates a mechanism by which a reduction in PSD-95 palmitoylation mediates chemically induced long-term depression (Chowdhury and Hell). Following calcium influx, binding of Ca^2+^/calmodulin to PSD-95 triggers a reduction in PSD-95 palmitoylation, and disperses the scaffolding protein and associated AMPARs out of the postsynaptic membrane. Remarkably, palmitoylation regulates PSD-95 conformation and orientation, thereby controlling the retention of AMPARs in PSDs (Jeyifous et al., 2016).

Protein palmitoylation also regulates the structural remodeling of neuronal processes. Albanesi et al. review the role of palmitoylation in the structural plasticity of dendritic spines. Spine remodeling depends on the polymerization, depolymerization, bundling, and branching of actin filaments. These processes are controlled by small GTPases and several other cytosolic proteins. Importantly, reversible palmitoylation enables their localization within spines in an activity-dependent manner.

Axonal growth and guidance are also regulated by protein palmitoylation. Dumoulin et al. report that protein palmitoylation plays a vital role in axonal bifurcation through activation of the signaling cascade triggered by the C-type natriuretic peptide and the receptor guanylyl cyclase Npr2 and cGMP-dependent protein kinase Iα.

In addition to synaptic membranes, palmitoylation also regulates the trafficking and functions of proteins in various organelles, including lysosomes. Sanders et al. have shown that palmitoylation regulates assembly of the mechanistic target of rapamycin (mTOR) complex 1 (mTORC1) signaling complex on lysosomal membranes. Interestingly, lysosomes translocate to dendritic spines and regulate local protein degradation, thereby facilitating synaptic remodeling (Goo et al., 2017). These findings suggest that the balance between palmitoylation and depalmitoylation regulates not only reversible trafficking but also proteostasis and autophagy.

A number of the palmitoylation and depalmitoylation enzymes are associated with various neurological and psychiatric disorders in children and adults. In Huntington disease, ZDHHC17 and ZDHHC13, also called huntingtin-interacting protein 14 (HIP14) and HIP14-like (HIP14L), directly interact with the huntingtin protein (Htt), and mutant Htt disrupts this interaction. Kang et al. find that HIP14L palmitoylates the NMDAR subunit GluN2B in striatal neurons. Consequently, deficient GluN2B palmitoylation enhances the extrasynaptic activity of NMDARs, which induces cell death-signaling pathways underlying neurodegeneration.

The lysosomal depalmitoylation enzyme PPT1 is associated with infantile neuronal ceroid lipofuscinosis. PPT1 is a crucial regulator of the autophagy-lysosome pathway and controls multiple steps of neuronal differentiation ranging from axonal guidance as well as pre- and post-synaptic functions (Koster and Yoshii). Especially, Sapir et al. identified the lack of PPT1 function results in immature dendritic spines and a reduction in LTP expression. Another recent study also demonstrates the loss of PPT1 function results in the stagnation of the developmental subunit switch of the NMDAR subunit that correlates with immature spine formation. Consequently, the NMDAR dysregulation underlies the susceptibility to excitotoxicity (Koster et al., 2019).

Progress in this field has been driven by new technical advantages that have allowed palmitoylation to be quantitatively studied, large numbers of substrates to be assayed, and the enzymes involved in the palmitoylation cycle identified. New tools for real-time analysis are finally becoming available. For example, it is now feasible to visualize the palmitoylated form of a particular protein (Gao and Hannoush, 2014) and assay depalmitoylation (Kathayat et al., 2017). Combining these techniques with super-resolution microscopy will enable live imaging of neuronal domains and organelles with new precision. Multi-photon microscopy has been invaluable in studying a wide range of structures from individual spines to neuronal circuitry. Applications of these new technologies will create exciting opportunities to tackle the above and other questions. New mechanistic details of about palmitoylation cycle and its role in synaptic development and plasticity should emerge and may provide insights into the pathology of various neurological and psychiatric disorders in children and adults.

## Author Contributions

All authors listed have made a substantial, direct and intellectual contribution to the work, and approved it for publication.

## Conflict of Interest

The authors declare that the research was conducted in the absence of any commercial or financial relationships that could be construed as a potential conflict of interest.
